# Endurance-training in healthy men is associated with lesser exertional breathlessness that correlates with circulatory-muscular conditioning markers in a cross-sectional design

**DOI:** 10.1186/2193-1801-3-426

**Published:** 2014-08-12

**Authors:** Laurent Plantier, Ghanima Al Dandachi, Cécile Londner, Aurore Caumont-Prim, Brigitte Chevalier-Bidaud, Jean-François Toussaint, François-Denis Desgorces, Christophe Delclaux

**Affiliations:** Physiologie Respiratoire – Clinique de la Dyspnée, Hôpital européen Georges-Pompidou, 20, rue Leblanc, Paris, 75015 France; AP-HP, Hôpital européen Georges-Pompidou, Unité d’Épidémiologie et de Recherche Clinique, Paris, 75015 France; Institut de Recherche bioMédicale et d’Epidémiologie du Sport, INSEP, Paris, France; Sorbonne Paris Cité, Faculté de médecine, Université Paris Descartes, Paris, 75006 France; Sorbonne Paris Cité, EA2511, Université Paris Descartes, Paris, 75014 France; CIC 9201 Plurithématique, Hôpital Européen Georges Pompidou, Paris, 75015 France; Assistance Publique-Hôpitaux de Paris, Hôpital Bichat-Claude Bernard, Service de Physiologie-Explorations Fonctionnelles, Paris, France, Université Paris Diderot, Paris, France, INSERM UMR700, Paris, France

**Keywords:** Dyspnoea, Exercise, Cardiopulmonary exercise test, Healthy man, Muscular conditioning, Physical activity

## Abstract

Whether exertional dyspnoea can be attributed to poor circulatory-muscular conditioning is a difficult clinical issue. Because criteria of poor conditioning such as low oxygen pulse, low ventilatory threshold or high heart rate/oxygen consumption slope can be observed in heart or lung diseases and are not specific to conditioning, we assessed the relationships between physical exercise, conditioning and exertional breathlessness in healthy subjects, in whom the aforementioned criteria can confidently be interpreted as reflecting conditioning.

To this end, healthy males with either low (inactive men, n = 31) or high (endurance-trained men, n = 31) physical activity evaluated using the International Physical Activity Questionnaire (IPAQ) underwent spirometry and incremental exercise testing with breathlessness assessment using Borg scale.

No significant breathlessness was reported before the ventilatory threshold in the two groups. Peak breathlessness was highly variable, did not differ between the two groups, was not related to any conditioning criterion, but correlated with peak respiratory rate. Nevertheless, endurance-trained subjects reported lower breathlessness at the same ventilation levels in comparison with inactive subjects. Significant but weak associations were observed between isoventilation breathlessness and physical activity indices (Borg at 60 L/min and total IPAQ scores, rho = -0.31, p = 0.020), which were mainly attributable to the vigorous domain of physical activity, as well as with conditioning indices (Borg score at 60 L.min^-1^ and peak oxygen pulse or heart rate/oxygen consumption slope, rho = -0.31, p = 0.021 and rho = 0.31, p = 0.020; respectively).

In conclusion, our data support a weak relationship between exertional breathlessness and circulatory-muscular conditioning, the later being primarily related to vigorous physical activity.

## Introduction

A common challenging issue in dyspnoea clinics is to determine whether dyspnoea can be attributed to reduced physical activity and subsequent poor circulatory-muscular conditioning in patients exhibiting normal or mildly altered exercise capacity, when cardio-pulmonary exercise testing fails to identify pathophysiological alterations consistent with respiratory or cardiovascular disease (Martinez et al. 1994).

Mechanisms driving breathlessness induced by physical exercise are highly complex and include multiple determinants such as metabolic needs, ventilatory demand, ventilatory capacity, cardiovascular response, peripheral neural afferences including mecanoreceptors and chemoreceptors, and cortical integration of signals involving emotional cues (Burki and Lee 2010). Interestingly, physical exercise itself holds an important place among therapeutical interventions known to reduce dyspnoea (breathlessness with negative affect) in patients with chronic heart or lung disease (Casaburi and ZuWallack 2009; Downing and Balady 2011), however the mechanisms of its effects deserve to be studied. In particular, whether better circulatory-muscular conditioning related to physical activity participates to a reduction in breathlessness/dyspnoea is unclear.

Conditioning comprises cardiac, microvascular and muscular adaptations which contribute to the optimization of oxygen delivery to exercising skeletal muscles. Markers of poor circulatory-muscular conditioning include slightly decreased peak oxygen consumption (  O_2_), early onset of the ventilatory threshold, an increased heart rate/  O_2_ (ΔHR/Δ  O_2_) relationship, and a reduction in oxygen pulse (American Thoracic Society and American College of Chest Physicians 2003; Palange et al. 2007). While determinants of conditioning are incompletely known, physical activity seems to play a major role, as improvements in the aforementioned criteria were demonstrated following exercise training over the whole range of human performance, from athletes undergoing intensive training to patients with severe obstructive lung disease (Laffite et al. 2003; Ramponi et al. 2013). While poor conditioning is commonly recognized as a possible cause of exercise intolerance or limitation (American Thoracic Society; American College of Chest Physicians 2003; Martinez et al. 1994; Palange et al. 2007), its participation in breathlessness/dyspnoea, although suspected (Parshall et al. 2012), deserves to be demonstrated. Data assessing relationships between conditioning/deconditioning criteria obtained from cardio-pulmonary exercise test and exertional breathlessness are scarce.

Criteria of poor conditioning such as low oxygen pulse, high ΔHR/Δ  O_2_ or low ventilatory threshold can be observed in heart or lung diseases and are thus not specific to conditioning, precluding the design of studies aiming at determining the impact of deconditioning on dyspnoea in diseased populations. For this reason, we chose to study the relationships between physical exercise, conditioning and exertional breathlessness in healthy subjects (without dyspnoea) with different levels of physical activity, in whom the aforementioned criteria could be confidently interpreted as reflecting conditioning. Consequently, indices of exertional breathlessness obtained from a cardiopulmonary exercise test (onset of breathlessness, isoventilation and iso  O_2_ comparisons, peak breathlessness) were compared in healthy men with either poor cardio-muscular conditioning (inactive) or good cardio-muscular conditioning (endurance-trained).

## Methods

### Subjects

Healthy (no medication, never smokers or ex-smokers < 5 pack-year, no history of asthma) Caucasian men were recruited from the Clinical Investigation Center of Pompidou hospital and sport clubs. The study population sample comprised 32 inactive subjects (i.e. no regular sport practice, < 1 hour/week for five consecutive years), between 20 and 60 years of age, and 32 endurance-trained subjects (sport practice > 3 hours/week for 5 consecutive years), between 20 and 60 years of age (see Table 1). Physical inactivity was defined as not meeting specified American physical activity guidelines of at least 1 hour and 15 minutes a week of vigorous-intensity aerobic physical activity ( http://www.health.gov/paguidelines/guidelines/summary.aspx). We used this definition because there is a lack of a clear and universal definition of a sedentary lifestyle (Varo et al. 2003). One inactive man was unable to perform lung function testing and serial breathlessness assessment was unavailable in one trained subject. These two men were excluded. This was a cross-sectional study in which informed written consent was obtained from all subjects, and ethical approval (Comité de Protection des Personnes Ile De France VI, ID-RCB: 2011-A00006-35) was received. The subjects have previously been described as this investigation constituted an ancillary study of the SAINVAPU trial devoted to explore the physiology of pulmonary vascular recruitment/dilation on exercise (Al Dandachi et al. 2014).

**Table 1:** **Subject characteristics**

Characteristic, median [25 ^th^ – 75 ^th^ percentiles]	Inactive men N = 31	Endurance-trained men N = 31	P Value
Age, years	37 [29–48]	41 [26–47]	0.714
Height, cm	177 [171–183]	180 [177–185]	0.051
Weight, kg	73 [69–85]	75 [69–84]	0.961
BMI, kg. m^2^	24.2 [22.5-25.6]	23.1 [21.8-24.9]	0.215
Rowing/Triathlon	0 / 0	19 / 12	<0.001
Sport practice, hours/week	0.00 [0.00-0.00]	7.00 [5.00-10.00]	<0.001
FEV_1_,% predicted	108 [100–115]	114 [106–123]	0.082
**Exercise test**			
Peak work rate, watts	201 [181–226]	318 [274–353]	<0.001
Peak work rate,% predicted	92 [80–97]	133 [120–144]	<0.001
Peak O_2_, L.kg.min^-1^	31.4 [28.5-37.4]	46.3 [39.6-52.6]	<0.001
Peak O_2_,% predicted	89 [75–101]	125 [114–136]	<0.001
Peak minute ventilation, L.min^-1^	86 [72–100]	127 [107–139]	<0.001
Peak minute ventilation, % predicted	88 [63–105]	110 [93–121]	0.001
Peak respiratory rate, cycles.min^-1^	34 [29–39]	44 [37–49]	0.001
Ventilatory capacity, %	61 [45–71]	77 [65–86]	<0.001
Peak heart rate, bpm.min^-1^	175 [162–183]	174 [168–183]	0.617
Δ O_2_/Δwork slope	9.3 [8.8-9.8]	9.4 [8.8-10.0]	0.468
Peak breathlessness, Borg	5.0 [2.5-7.0]	7.0 [4.0-8.0]	0.570
Peak leg discomfort, Borg	7.0 [4.0-9.0]	5.0 [4.0-8.0]	0.233
Borg score at 30% E_max predicted_	2.0 [1.0-2.5]	1.0 [0.5-2.0]	0.020
*Power Law of* *O*_*2*_ *-Breathlessness*			
Constant a	0.087 [0.002-0.650]	0.001 [0.00001-0.202]	0.035
Exponent b	2.69 [2.05-3.56]	2.84 [2.10-4.35]	0.548

BMI denotes body mass index.

### Physical activity evaluation

Physical activity was evaluated using the French version of the International Physical Activity Questionnaire (IPAQ, long form) (Macfarlane et al. 2006) administered by the physician in charge of the exercise test, before testing. IPAQ comprehensively assesses physical activity performed during leisure time physical activity, domestic and gardening activities, work-related physical activity, and transport-related physical activity (Craig et al. 2003). The items in the long IPAQ form were structured to provide separate domain-specific scores for walking, moderate-intensity, and vigorous-intensity activity presented as median MET–minutes/week. The mean number of hours per week spent on sport practice was also recorded as a crude indicator of vigorous activity.

### Spirometry and cardio-pulmonary exercise test

Spirometry was performed on MasterScreen systems (Jaeger, Carefusion, San Diego, USA), using reference values published by Quanjer and colleagues (Quanjer et al. 1993). Symptom-limited incremental exercise testing was conducted on an electronically braked cycle ergometer using the Vmax Cardiopulmonary Exercise Testing System (Sensor Medics, Yorba Linda, CA) according to the recommended guidelines and reference values (American Thoracic Society; American College of Chest Physicians 2003), as previously described (Delclaux et al. 2011). After a 2 min warm-up period (inactive: 30 watts; trained subjects: 50 watts), the workload was increased by 15–30 Watts/minute, according to the degree of fitness, using a ramp protocol until exhaustion in order to obtain similar durations of exercise for the two groups of subjects. Ventilation and gas exchange measurements were performed breath-by-breath. Subjects rated the magnitude of their perceived breathing (breathlessness without negative affect) and leg discomfort at rest, every minute during exercise and at peak exercise by pointing to the 10-point Borg scale (Category scale with Ratio properties: CR10 (Borg and Kaijser 2006), explained before testing); zero represented "no discomfort" and 10 represented "the most severe discomfort they could imagine experiencing". Slopes of Δ  O_2_/ΔWork rate, ΔHR/Δ  O_2_, Δ minute ventilation (  E) /Δ  O_2_, Δ  E / Δ carbon dioxide production (  CO_2_), and the anaerobic threshold (ventilatory threshold, using noninvasive determinations: ventilatory equivalents and V slope methods (American Thoracic Society; American College of Chest Physicians 2003) were calculated. Pa_CO2_ was monitored continuously using transcutaneous measurement at the ear (TOSCA 500, Radiometer, Copenhagen, Denmark), which allowed for the calculation of the physiological dead space/ tidal volume (VD/VT) ratio (corrected for valve dead space), as previously described (Delclaux et al. 2011). Percentage of predicted peak minute ventilation was calculated as 100 × peak  E / 35 × forced expiratory volume in 1 second (FEV_1_).

Discomfort (leg, breathlessness) evaluation. In order to obtain an index characterising breathlessness during exercise related to ventilation available for all subjects, the Borg breathlessness score corresponding to 30% of predicted maximal ventilation was calculated. We also characterised the  O_2_ /breathlessness or  O_2_ /leg discombort relationships according to a power law: Breathlessness or leg discomfort = a ×  O_2_^b^ (Delclaux et al. 2011).

### Statistics

Data are given as median, lower and upper quartiles. Comparisons between groups were performed using Mann-Whitney’s U-test. Univariate correlations were estimated using non parametric Spearman coefficients. Multivariate analyses were performed using multivariate regression (backward) after log-transformation of the variables. A p value < 0.05 was deemed significant. Statistical analyses were performed using Statview 5.0 software (SAS Institute, Inc, Cary, NC, USA). Due to the exploratory design of the study, no correction for multiplicity of testing was done (Rothman 1990).

## Results

**Figure 1:**
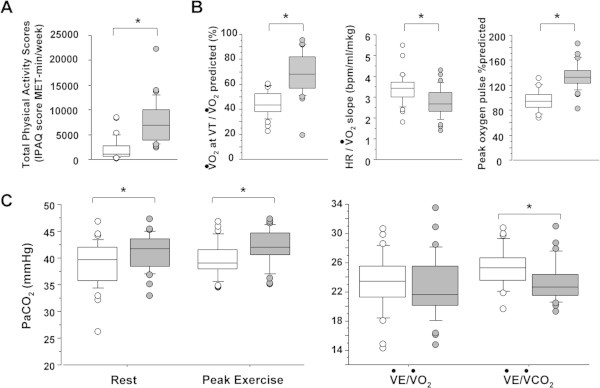
**Description of physical activity, conditioning markers and ventilatory demand in inactive and endurancetrained men. A**] Total physical activity scores (total IPAQ, see Methods), **B**] Circulatory-muscular conditioning markers (  O2 at ventilatory threshold (VT), heart rate (ΔHR) / Δ  O_2_ slope, peak oxygen pulse) and **C**] PaCO_2_ and ventilatory efficiency (Δ  E/Δ  O2 and Δ  E/Δ  CO_2_ slopes) markers in inactive (n = 31, white symbols) and endurance-trained (n = 31, grey symbols) men. Data are shown as medians, interquartile range, and extreme values. *p < 0.05.

As shown in Table 1, inactive and endurance-trained subjects were well matched anthropomorphically. Similar peak heart rates were obtained in the two groups. The reasons for stopping the effort were similar in inactive and trained men (leg discomfort: 23 versus 21; breathlessness: 3 versus 6 and both: 5 versus 4; p = 0.587). The median IPAQ score of inactive and endurance-trained subjects was 1099 [695–2687] and 6966 [3865–10005] MET-min/week, respectively (Figure 1A). A close correlation between the number of hours spent on sport and the vigorous-activity domain of IPAQ was observed (Rho = 0.886, p < 0.001).

As expected, endurance-trained subjects had better performance indices with higher peak work rate and peak  O_2_. Likewise, all indices of conditioning (  O_2_ at ventilatory threshold, oxygen pulse, ΔHR/Δ  O_2_ slope) were significantly different in endurance-trained compared to inactive subjects, consistent with increasing circulatory-muscular conditioning in the former group (Figure 1B). Endurance-trained subjects had slightly increased PaCO_2_ in comparison with inactive subjects, both at rest and peak exercise, as well as better ventilatory efficiency as shown by decreased Δ  E/Δ  CO_2_ slope (Figure 1C). Physiological VD/VT ratio was very similar in both groups (0.34 [0.28-0.38] versus 0.34 [0.32-0.38], p = 0.472).

In both inactive and endurance-trained men, no significant breathlessness was reported before the ventilatory threshold (Borg score at ventilatory threshold: 0.5 [0–1] in inactive men, 0.5 [0–2] in endurance-trained men, p = 0.413). Peak breathlessness or perceived leg discomfort Borg scores did not differ between the two groups, and showed considerable variability (Table 1). Peak breathlessness was not related to performance (% predicted  O_2_) nor with any conditioning criterion. Among physiological parameters assessing ventilatory demand or response, peak breathlessness positively correlated with peak respiratory rate (rho = 0.301, p = 0.020).

**Figure 2:**
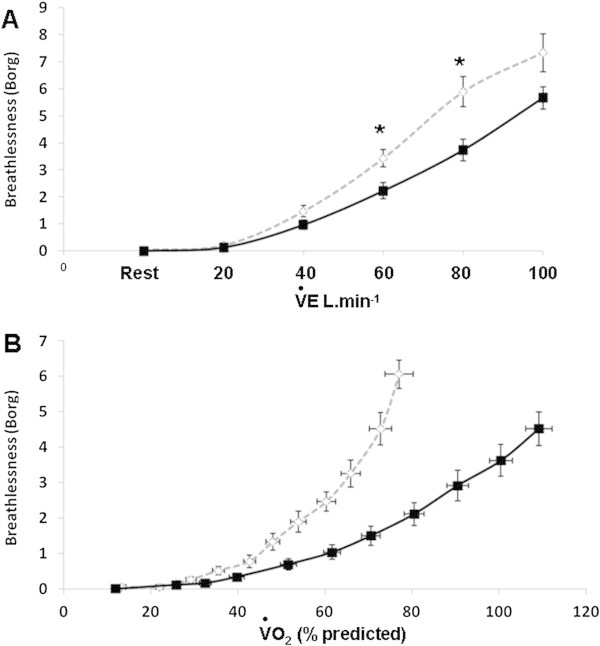
**Relationships between breathlessness assessed by Borg scale and A] minute ventilation (**  **E) expressed as absolute value and B]**  **O**_**2**_ **expressed as a percentage of predicted value, in inactive men (n = 31, empty circles and dotted lines) and endurance-trained men (n = 31, filled squares and continuous lines).** Upper panel. It gives the level of breathlessness (Borg score) according to the level of ventilation (20 to 100 L.min^-1^). Each point for a single patient is calculated by linear interpolation since the degree of breathlessness during the CPET is obtained according to time. All subjects did not reach  E at 60 L.min^-1^. Lower panel. Mean values of  O_2_ and Borg score (obtained by interpolation) are given every minute of the exercise test in the two groups of subjects. Data are shown for a minimum of n = 10 subjects at each  O_2_ point. Mean values and standard error of the mean are represented. *p < 0.05 between inactive and endurance-trained men.

Borg breathlessness scores were then compared at isoventilation levels. As shown in Figure 2A, endurance-trained subjects reported lower breathlessness at 60 and 80 L.min^-1^ of ventilation, in comparison with inactive subjects. Similarly, the perceived leg discomfort was reduced in the more active group at 40, 60, 80 and 100 L.min^-1^ (p < 0.001, p < 0.001, p = 0.002, p = 0.006, respectively).

The Borg breathlessness score corresponding to 30% of predicted maximal ventilation (see Table 1), characterizing the upper shift shown in Figure 2A, was logically significantly higher in inactive men as compared to trained men. Reduced breathlessness for similar exercise intensity in endurance-trained subjects was also readily apparent when breathlessness was plotted against  O_2_ (Figure 2B).

In line with these observations, the modelling of the  O_2_ /breathlessness relationship according to a power law showed a higher "a" constant in inactive men as compared to trained men (Table 1). Then, the exponent b was calculated for the  O_2_ /perceived leg discomfort relationship. The ratio of the exponents (b) was 0.95 ± 0.34 (  O_2_ / breathlessness versus  O_2_ / leg discomfort), which was not significantly different from 1, suggesting similar shapes of increment on exercise of both breathlessness and leg discomfort.

**Figure 3:**
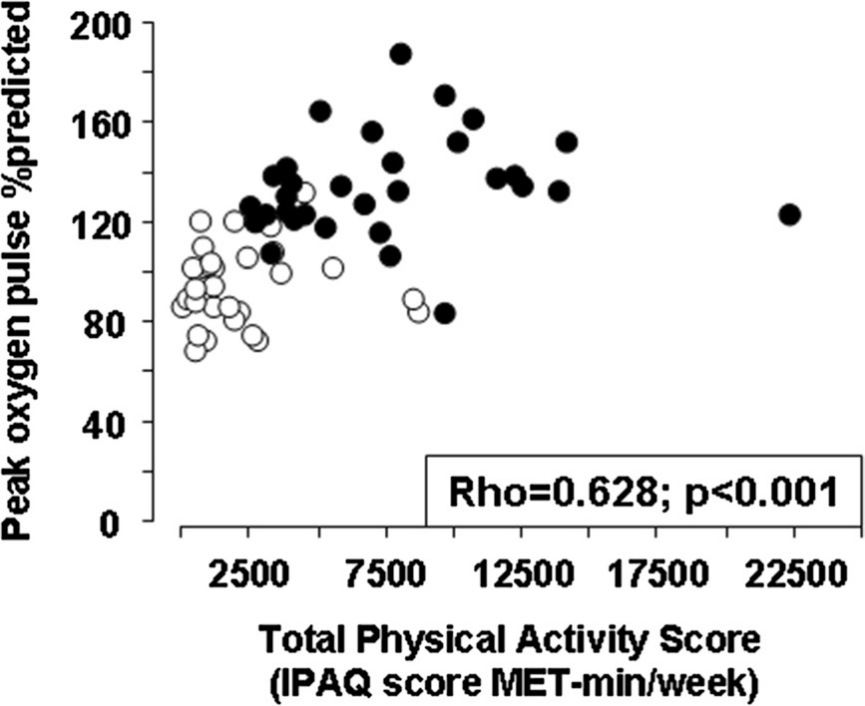
**Peak oxygen pulse expressed as percentage of predicted value, plotted against total physical activity score (IPAQ, see Methods), in inactive (n = 31, empty circles) and endurance-trained men (n = 31, filled circles).**

Because both performance and conditioning indices showed overlap between inactive and endurance-trained subjects, data from the two groups were pooled to further explore relationships between activity levels, conditioning indices, and breathlessness. To determine whether conditioning indices were related to the level of physical activity, we explored the correlations between the total IPAQ score and its determinants on the one hand, and  O_2_ at Ventilatory Threshold, ΔHR/Δ  O_2_ slope and the oxygen pulse on the other hand, as well as age. As shown in Table 2 and Figure 3A, significant correlations were observed between all conditioning indices, peak  O_2_, and total physical activity. However, whereas moderate and vigorous physical activities were strongly associated with indices of circulatory/muscular conditioning, no association was observed between walking index and conditioning. Age and BMI were not associated with any conditioning index.

**Table 2:** **Univariate spearman analysis of conditioning marker correlates**

Total n = 62	Age	IPAQ total	IPAQ walking	IPAQ moderate	IPAQ vigorous
ΔHR/Δ O_2_	P = 0.703	P = 0.001	P = 0.281	P < 0.001	P = 0.005
Slope	Rho = -0.049	Rho = -0.426	Rho = -0.139	Rho = -0.453	Rho = -0.359
Peak O_2_ pulse	P = 0.443	P < 0.001	P = 0.852	P = 0.011	P < 0.001
(% predicted)	Rho = 0.098	Rho = 0.628	Rho = 0.024	Rho = 0.325	Rho = 0.703
Ventilatory	P = 0.514	P < 0.001	P = 0.317	P = 0.072	P < 0.001
Threshold*	Rho = 0.084	Rho = 0.461	Rho = -0.128	Rho = 0.232	Rho = 0.584
Peak O_2_	P = 0.510	P < 0.001	P = 0.723	P = 0.012	P < 0.001
(% predicted)	Rho = 0.086	Rho = 0.588	Rho = -0.045	Rho = 0.321	Rho = 0.663

The table describes the statistical correlations (Spearman coefficient: Rho and the P value of the test) between the conditioning markers (first column) and age or the different domains of IPAQ (different levels of physical activity).

*expressed as% of peak  O_2_ predicted.

**Figure 4:**
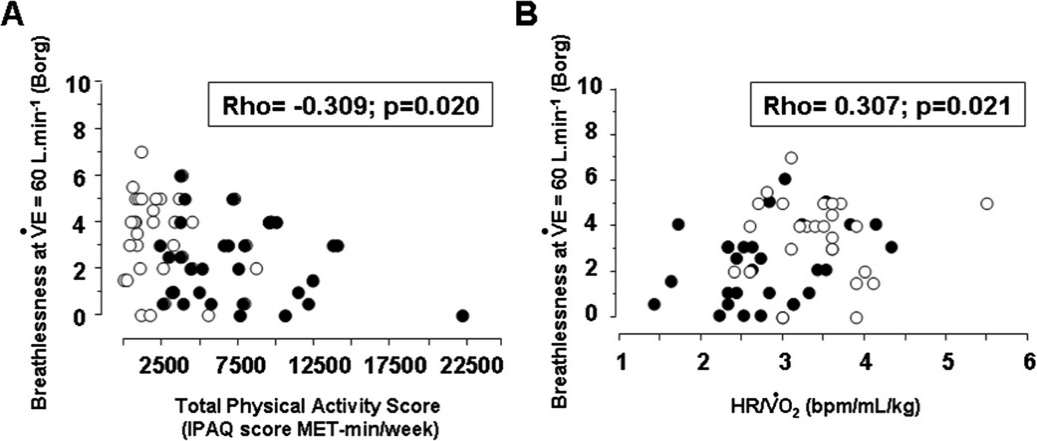
**Breathlessness at 60 L.min** ^**-1**^ **ventilation plotted against A] total physical activity score (IPAQ score, see Methods) and B] heart rate (ΔHR)/ Δ**  **O**_**2**_ **,** **in inactive (n = 31, empty circles) and endurance-trained men (n = 31, filled circles).**

Because endurance-trained subjects showed reduced exertional breathlessness in comparison to inactive subjects at isoventilation levels of 60 L.min^-1^ and 80 L.min^-1^, we then aimed to determine whether breathlessness at these ventilation levels (and also for normalized ventilation at 30%  E maximal predicted) was correlated with physical activity levels, conditioning indices, or ventilatory efficiency. As shown in Table 3, univariate correlation analyses showed significant associations between breathlessness at isoventilation levels and physical activity (Figure 4A), which were mainly attributable to the vigorous domain of physical activity. Significant associations were also found between exertional breathlessness and indices of cardio-muscular conditioning (Figure 4B). By contrast, ventilatory efficiency/demand (Δ  E/Δ  CO_2_ slope) was not associated with these breathlessness criteria in this cohort of subjects free of any lung or heart disease. Age was not associated with any breathlessness index.

**Table 3:** **Relationships between breathlessness indices and conditioning or ventilatory demand**

Total N = 62	IPAQ total	IPAQ walking	IPAQ moderate	IPAQ vigorous	ΔHR/Δ O _2_ slope	Peak O _2_ pulse (% predicted)	Ventilatory threshold*	Δ E/Δ CO _2_ slope
Borg-60	P = 0.0198	P = 0.6623	P = 0.3692	P = 0.0238	P = 0.0199	P = 0.0206	P = 0.1656	P = 0.8112
L.min^-1^	Rho = -0.309	Rho = 0.058	Rho = -0.119	Rho = -0.299	Rho = 0.311	Rho = -0.307	Rho = -0.184	Rho = -0.032
Borg-80	P = 0.0138	P = 0.7169	P = 0.1537	P = 0.0296	P = 0.0290	P = 0.0279	P = 0.1667	P = 0.8402
L.min^-1^	Rho = -0.380	Rho = -0.056	Rho = -0.220	Rho = -0.336	Rho = 0.341	Rho = -0.339	Rho = -0.213	Rho = 0.031
Borg-30%	P = 0.0194	P = 0.958	P = 0.379	P = 0.021	P = 0.012	P = 0.031	P = 0.027	P = 0.725
E_max_ _pred_	Rho = -0.299	Rho = 0.007	Rho = -0.113	Rho = -0.294	Rho = -0.320	Rho = -0.256	Rho = -0.284	Rho = -0.045

The table describes the statistical correlations (Spearman coefficient: Rho and the P value of the test) between isoventilation breathlessness indices (first column) and the different domains of IPAQ or conditioning markers or ventilatory demand (Δ  E/Δ  CO_2_ slope).

*expressed as% of peak  O_2_ predicted.

The association of breathlessness at isoventilation levels with parameters related to physical activity and cardio-circulatory conditioning was then tested using multivariate analyses. These analyses confirmed that physical activity and conditioning indices were not independent determinants of breathlessness.

## Discussion

The main results of this cross-sectional physiological study are that 1) the onset of breathlessness began concomitantly with a conditioning criterion (ventilatory threshold), 2) breathlessness and perceived leg discomfort had similar shapes of increment on exertion, and 3) trained subjects experienced less breathlessness for similar levels of ventilation than inactive men, which was associated with the degree of conditioning. The three results suggest a weak but significant link between circulatory-muscular conditioning and exertional breathlessness, at least in healthy men.

The link between peak  O_2_ and physical activity in our study is in line with previous studies showing significant relationships between leisure time physical activities and aerobic capacity in healthy subjects, where such associations were mostly driven by high-intensity activities and independent of age (Loe et al. 2013; Talbot et al. 2000). Our observation of correlations between physical activity on the one hand, and ΔHR/Δ  O_2_, oxygen pulse and the ventilatory threshold on the other hand, support the hypothesis that such parameters may be considered physiological markers of circulatory-muscular conditioning as suggested by ATS guidelines (American Thoracic Society; American College of Chest Physicians 2003). However, although relationships between physical activity and conditioning markers were statistically significant in our study, Rho values indicated mostly moderate relationships, in agreement with previous studies where  O_2_-activity relationships were weak (Loe et al. 2013). This finding is consistent with the fact that peak  O_2_ and peak oxygen pulse are firmly related to genetic hereditary, as was demonstrated in studies in siblings and in dizygotic and monozygotic twins, where genetic factors accounted for 40% of variance in peak  O_2_ and 60% of variance in oxygen pulse (Bouchard et al. 1986).

In addition to increased peak  O_2_ and markers of circulatory-muscular conditioning, endurance-trained men showed a decreased Δ  E/Δ  CO_2_ slope (ventilatory demand) in comparison with inactive men indicating better ventilatory efficiency, consistent with a beneficial effect of training regarding  E/  CO_2_ (Yerg et al. 1985). Because physiological dead space measurements were very similar in both groups, this increase in ventilatory efficiency can be attributed to the relative alveolar hypoventilation observed in endurance-trained subjects. This result raises the hypothesis that elevation of the PaCO_2_ set-point may be a usual adaptation to endurance training. The mechanisms by which training may alter the PaCO_2_ set-point are unclear, as no difference in CO_2_ chemosensitivity has been found between endurance-trained athletes and control subjects (McConnell and Semple 1996). Since PaCO_2_ did not correlate with physical activity levels, whether hypoventilation was related to training or to selection bias cannot be inferred from our results.

Our data support a relationship between endurance training, circulatory-muscular conditioning and exertional breathlessness. Firstly, breathlessness began at the ventilatory threshold, which is tightly related to conditioning. Secondly, breathlessness and perceived leg discomfort muscular fatigue developed in a similar way (similar b exponents), and thirdly trained subjects showed reduced breathlessness at similar levels of ventilation in comparison with inactive subjects with significant correlations between isoventilation breathlessness, physical activities and conditioning indices. Decreased breathlessness at isoventilation in trained subjects could be related to better conditioning of respiratory muscles (greater inspiratory muscle strength), higher ventilatory capacity and/or desensitization to the stimulus. The perceived leg discomfort/fatigue was similarly reduced in the more active group suggesting greater global skeletal muscle strength or desensitization. In support of the former hypothesis, Hamilton and colleagues showed a relationship between power output measured during an exercise test and dyspnoea, according to the strength of inspiratory muscles (Hamilton et al. 1995). In support of the latter, physical activity, as performed in the setting of rehabilitation programs, has been shown to decrease exertional dyspnoea in patients with heart or respiratory diseases, even in the absence of a benefit in terms of performance (Casaburi and ZuWallack 2009), while repetition of exercise tests in patients with obstructive lung disease has also been associated with short-term reduction in breathlessness (Belman et al. 1991).

Despite a correlation between breathlessness and respiratory rate at peak exercise, our results show a partial disconnect between breathlessness and respiratory effort, in line with concepts linking exertional breathlessness or dyspnoea to multiple determinants beyond an increased work of breathing (Burki and Lee 2010). Whether altered PaCO_2_ set-point or CO_2_ chemosensitivity contributed to reduced exertional breathlessness in trained subjects, or vice versa, could not be evidenced in this study.

Our study has inherent limitations due to its cross-sectional design. Statistical differences and correlations did not demonstrate causality. Reporting bias may be suspected as physical activity levels were measured using questionnaires (Macfarlane et al. 2006), whereas over-reporting of physical activity in males has been described (Klesges et al. 1990). Nevertheless, IPAQ long form has been recommended for research requiring more detailed assessment (Craig et al. 2003). As only men were included, results may not be valid in women. All subjects were healthy and did not report dyspnoea, defined by a negative affect associated with breathlessness, mandating caution before extrapolation of our results to diseased populations. Finally, because vigorous exercise was inherently rare in inactive patients, selection bias may have led to overestimation of its weight relative to moderate exercise or walking.

From a clinical perspective, our results raise two important issues. First, in what measure can excessive exertional breathlessness be attributed to poor conditioning in an inactive or sedentary patient with a complaint of dyspnoea? Since statistical relationships between isoventilation breathlessness and conditioning criteria were weak (~10% of breathlessness variance), and due to the multiplicity of determinants of physiological breathlessness, in clinical practice it may be very difficult to confidently attribute dyspnoea to circulatory-muscular deconditioning in otherwise healthy subjects, a diagnosis that may be overestimated.

Second, in these results obtained in healthy men, and in agreement with a previous study (Talbot et al. 2000), walking or moderate physical activity played a minor role in comparison with vigorous physical activity in terms of increased circulatory-muscular conditioning and reduced exertional breathlessness, raising the question whether rehabilitation programs offered to patients with heart or respiratory failure may be improved by a focus on high-intensity, high breathlessness exercise as suggested by the recent demonstrations of superiority of nonlinear or interval exercise training in patients with chronic obstructive pulmonary disease or heart failure (Haykowsky et al. 2013; Klijn et al. 2013).

In conclusion, cardio-muscular conditioning indices and an index of exertional breathlessness are linked to a weak extent in healthy men. As a consequence, in otherwise healthy patients it may be difficult to ascribe dyspnoea to circulatory-muscular deconditioning.

## Abbreviations

(IPAQ): International Physical Activity Questionnaire
(VD/VT): dead space/tidal volume
(FEV_1_): forced expiratory volume in 1 second
(BMI): body mass index
